# A Comprehensive review of abdominal infections

**DOI:** 10.1186/1749-7922-6-7

**Published:** 2011-02-23

**Authors:** Nicole Lopez, Leslie Kobayashi, Raul Coimbra

**Affiliations:** 1 200 West Arbor Dr. #8896, San Diego CA 92103-8896, USA; 2 Assistant Professor of Surgery, University of California, San Diego, 200 W. Arbor Dr. #8896, San Diego, CA 92103-8896, USA; 3 Monroe E. Trout Professor of Surgery, Executive Vice-Chairman, Department of Surgery, Chief, Division of Trauma, Surgical Critical Care, and Burns, University of California, San Diego, 200 West Arbor Dr., #8896, San Diego, CA 92103-8896, USA

## Introduction

Intra-abdominal infection (IAI) is an important cause of morbidity and mortality. It is the second most commonly identified cause of severe sepsis in the intensive care unit (ICU). Recent studies have associated severe intra-abdominal infection with a significant mortality rate.

Most IAI are a result of processes involving inflammation and perforations of the gastrointestinal tract, such as appendicitis, peptic ulcer disease, and diverticulitis. Patients with diffuse peritonitis may be due to spontaneous perforation, post-operative, post-interventional or post-traumatic causes. The lower GI tract is most often the location of perforation. Among patients with IAI who develop peritonitis, many may progress to severe sepsis, defined by The American College of Chest Physicians/Society of Critical Care Medicine as a severe systemic inflammatory response to infection that is associated with acute organ dysfunction.

Successful treatment of IAI is based on early and appropriate source recognition, containment and antimicrobial coverage. We will review clinical definitions, pathophysiology, and treatment strategies for IAI in an effort to provide guidelines for clinical management.

## Definitions

**Intra-abdominal infection (IAI) **describes a diverse set of diseases. It is broadly defined as peritoneal inflammation in response to microorganisms, resulting in purulence in the peritoneal cavity[1]. IAI are classified as uncomplicated or complicated based on the extent of infection[2].

**Uncomplicated abdominal infections **involve intramural inflammation of the gastrointestinal (GI) tract without anatomic disruption. They are often simple to treat; however, when treatment is delayed or inappropriate, or the infection involves a more virulent nosocomial microbe, the risk of progression into a complicated abdominal infection becomes significant[3,4].

**Complicated abdominal infections **extend beyond the source organ into the peritoneal space. They cause peritoneal inflammation, and are associated with localized or diffuse peritonitis[5]. Localized peritonitis often manifests as an abscess with tissue debris, bacteria, neutrophils, macrophages, and exudative fluid contained in a fibrous capsule. Diffuse peritonitis is categorized as primary, secondary or tertiary peritonitis.

**Primary peritonitis **is also known as spontaneous bacterial peritonitis. It is thought to be the result of bacterial translocation across an intact gut wall[6]. These infections are commonly monomicrobial, and the infecting organism is primarily determined by patient demographics. For example, healthy young girls are most often infected by streptococcal organisms, cirrhotics by gram negative or enterococcal organisms, and peritoneal dialysis patients by *Staphylococcus aureus*[7,8]. Diagnosis requires peritoneal fluid aspiration. Characteristics of infection include white blood cell count (WBC) > 500 cells/mm^3^, high lactate, and low glucose levels. Positive peritoneal fluid cultures are definitive, and resolution of infection is marked by peritoneal fluid with < 250 WBC/mm^3^[9].

**Secondary peritonitis **is caused by microbial contamination through a perforation, laceration, or necrotic segment of the GI tract[7]. Definitive diagnosis is based on clinical examination and history, and specific diagnoses can be confirmed by radiographic imaging[10]. If a patient is stable enough for transport, computed tomography (CT) scan with intravenous and oral contrast is the standard method of evaluating most intra-abdominal pathologies, such as appendicitis, diverticulitis, and colitis[11]. Suspected biliary pathology is the exception, and ultrasound is the preferred initial imaging modality for this spectrum of disease including acute cholecystitis, emphysematous cholecystitis, and cholangitis. Infections associated with secondary peritonitis are commonly polymicrobial and the infecting organisms are those most commonly associated with the source of contamination (see Table 1).

**Table 1:** Expected organisms according to source

	Source	Expected Organism
**Primary Peritonitis**	Young healthy female	Streptococcus
	Cirrhotic	Enteric gram negatives Enterococcus
	CAPD	Staphylococcus aureus
**Secondary peritonitis**	Stomach and duodenum	Streptococcus Lactobacillus
	Biliary	E. coli, Klebsiella, Enterococcus
	Small Intestine	E. coli, Klebsiella, Lactobacillus Streptococci Diptheroids Enterococci
	Distal ileum and colon	Bacteroides fragilis Clostridium spp. E. coli Enterobacter spp. Klebsiella spp. Peptostreptococci Enterococci
**Teritiary peritonitis**		Enterococcus Candida Staphylococcus epidermidis Enterobacter

Adapted from Weigelt JA [12].

**Tertiary peritonitis **represents an infection that is persistent or recurrent at least 48 hours after appropriate management of primary or secondary peritonitis. It is more common among critically ill or immunocompromised patients[12]. Because of the poor host defenses, it is also often associated with less virulent organisms, such as *Enterococcus*, *Candida*, *Staphylococcus epidermidis*, and *Enterobacter*[13].

**Intra-abdominal sepsis **is an IAI that results in severe sepsis or septic shock[2].

## Pathophysiology

The peritoneum divides the abdomen into the peritoneal cavity and the retroperitoneum. The peritoneum is a layer of mesothelium that lines the abdominal cavity. It is abundantly innervated by the somatic nervous system. This explains the intense localized pain that patients experience when they have peritoneal inflammation or injury. Functionally, it provides approximately one m^2 ^of exchange area, and holds approximately 100 ml of peritoneal fluid, primarily consisting of macrophages and lymphocytes[14,15]. Negative pressure generated by diaphragmatic relaxation causes peritoneal fluid to flow upward toward a specialized system of diaphragmatic fenestrae. This high flow system drains fluid into the lymphatic system. During infection, this allows for rapid efflux of micro-organisms and host defenses into the venous system via the thoracic duct[16].

Perforation, and the bacterial innoculation that ensues, causes an inflammatory response that acts locally to contain the infection; but, in the setting of overwhelming contamination, it can spread to cause systemic inflammation.

Several mechanisms act locally to contain or destroy infection. Tissue injury stimulates mast cell degranulation. Mast cell degranulation releases histamine, kinins, leukotrienes, prostacyclines, and free radicals. These factors increase vascular and peritoneal permeability allowing for local influx of complement and coagulation cascade factors.

Influx of complement at the site of contamination allows for bacterial opsonization via C3b. Diaphragmatic motion, described above, then leads to absorption of bacteria laden peritoneal fluid into the lymphatic system. Opsonised organisms in the lymph are transported to the reticuloendothelial system, where they are destroyed. In addition to bacterial destruction via opsonization, complement also attracts neutrophils to the site of injury via chemotactic factors C3a and C5a. Neutrophils attack bacteria by three mechanisms: first they express and release more cytokines further propagating the inflammatory response; second, they phagocytose and destroy bacteria via respiratory burst; third they secrete neutrophil extracellular traps (NETs). NETs are composed of DNA, chromatin and serine proteases. NETs can both destroy extracellular organisms without phagocytosis, and act as a physical barrier to prevent the further spread of pathogens[17]. Finally, tissue factor, expressed by injured tissue, leads to activation of the coagulation cascade. This results in increased fibrin production, necessary to contain bacteria by abscess formation.

These cellular processes can also have systemic effects, as the products of mast cell degranulation at the site of injury move into the circulatory system. There, in addition to increased vascular permeability, they cause smooth muscle relaxation and can result in peripheral vascular collapse. Free radicals released with degranulation cause lipid peroxidation of cell membranes resulting in further release of toxic granulation products. Granulocytes and macrophages, attracted to the site of injury by the complement chemotactic factors C3a and C5a, release acute phase cytokines such as IL-1, IL-6, TNF-α, IFN-γ. These cytokines are released into the peripheral circulation where they cause fever, cortisol release, acute phase protein synthesis, leukocytosis, and lymphocyte differentiation and activation. The resultant physiologic state is clinically known as the Systemic Inflammatory Response Syndrome (SIRS). SIRS is defined by the presence of at least two of the following: core body temperature > 38°C or < 36°C, heart rate > 90 beats per minute, respiratory rate > 20 breaths per minute (not ventilated) or PaCO_2 _< 32 mmHg (ventilated), WBC > 12,000, < 4,000, or > 10% immature forms (bands)[18]. When SIRS is associated with a bacterial source, as with cases of IAI, it is known as sepsis. When sepsis is paired with organ failure, it is known as severe sepsis.

## Management

Management of IAI requires resuscitation, source control, and antibacterial treatment. The most important of these factors is source control, which, "encompasses all measures undertaken to eliminate the source of infection and to control ongoing contamination"[19]. There are three key components of source control: drainage, debridement, and definitive management.

### Resuscitation and Support of Organ Systems

IAI causes volume depletion by several mechanisms. Nausea, anorexia and ileus lead to a decrease in oral intake, while vomiting and diarrhea increase sensible losses. In addition, ileus with third space losses into the bowel wall and ascites, as well as fever both increase insensible losses. Elevated body temperature leads to both an increase in dermal loss via sweating, and an increase in respiratory loss by causing tachypnea. Dermal loss in a febrile patient can account for approximately 600 ml of volume loss per day, while tachypnea causes approximately 100 ml of volume loss per day[20,21].

In uncomplicated IAI, replacing volume is essential; in severe sepsis or septic shock, it becomes critical. Patients suspected of having severe sepsis or septic shock should be admitted to an ICU for careful monitoring of vital signs and volume status. With regard to the initial volume resuscitation, we recommend following the Surviving Sepsis Campaign recommendations. As soon as hypotension is recognized, or, ideally if it is anticipated, attention should be paid to early goal directed volume resuscitation. Isotonic fluid, or in the cases of severe anemia or coagulopathy, blood products, should be administered with the intent to achieve a mean arterial pressure (MAP) > 65 mmHg and a central venous pressure (CVP) of 12-15 mmHg within the first 6 hours[22]. If a MAP > 65 mmHg cannot be obtained by volume resuscitation alone then vasopressors should be used, with a preference for norepinepherine or dopamine[22]. In cases where low cardiac output or elevated filling pressures indicate severe myocardial dysfunction, use of inotropic agents such as dobutamine may be efficacious in obtaining adequate MAP[22]. Care should also be taken to monitor clinical indicators of end organ perfusion, such as hourly urine output and mental status, to ensure adequate oxygen delivery.

The goal of resuscitation is correction of cellular oxygen debt. Various endpoints for resuscitation have been suggested, including: mixed venous oxygen (SVO_2_), lactate and base deficit. While a normal or high SVO_2 _does not ensure adequate tissue oxygenation, a low SVO_2 _indicates a need to increase tissue oxygenation. Resuscitation to maintain an SVO_2 _> 65% has been shown to improve outcomes[23,24]. Lactate, a product of anaerobic metabolism, has also been used as an indirect measure of oxygen debt. More recently sepsis has been recognized as a hypermetabolic state that uses glycolysis in the absence of hypoxia, making it less reliable as a marker of oxygen debt. Still, its early normalization may predict improved outcomes[25-27]. Base deficit is yet another indicator of oxygen debt. It describes the amount of base that would be required to bring the blood to a normal pH under normal physiologic conditions. The degree of base deficit has been shown to correlate with resuscitation requirements and mortality[28,29]. While none of these measures are perfect, they can be helpful in guiding resuscitation when used in combination with the other clinical endpoints discussed above.

### Drainage

The goal of drainage is to evacuate purulent, contaminated fluid, or to control drainage of ongoing enteric contamination. This is accomplished by either percutaneous or open surgical intervention. Percutaneous drainage can be performed with or without image guidance, and is most commonly performed using ultrasound or CT. In many circumstances it is as efficacious as surgical drainage, and is often used as the initial treatment of choice because it is less invasive and more affordable[30,31]. Percutaneous drainage is also useful in patients who are poor surgical candidates, and might not survive definitive surgical treatment. However, percutaneous drainage is unlikely to result in adequate source control in cases of frank bowel perforation with ongoing contamination, or if there is a significant amount of necrotic tissue present. In these cases, surgery is the treatment of choice.

Open surgical drainage should be used in the case of generalized peritonitis, ongoing gross contamination from an uncontrolled enteric source, if bowel necrosis or ischemia is suspected, and in cases of failure of percutaneous drainage. Unstable patients, or those with complicated or difficult anatomy such as post-operative patients or those with advanced malignancy pose a particular challenge.

In these situations, damage control techniques can be employed with temporary abdominal closure. Damage control procedures are typically used for patients who are unstable and unable to tolerate definitive surgical treatment, have intra-abdominal hypertension (IAH), or have loss of abdominal domain that prevents fascial closure. The first stage in damage control surgery is evacuation of infected material and control of gross contamination. This is followed by temporary abdominal closure with a conventional dressing, negative pressure dressing, or skin closure. This first operative stage is followed by ongoing resuscitation, once normal physiology is restored resuscitation can then be followed by planned re-laparotomy for definitive source control and reconstruction. In cases of physiologic worsening after first laparotomy, or in cases of concern for IAH, or intestinal ischemia, on demand repeat laparotomy can be performed. Once all surgical issues have been addressed, physiology has been restored and there are no longer concerns for ongoing ischemia, necrosis, or IAH the abdomen can be definitively closed.

Intra-abdominal lavage is a subject of ongoing controversy. Proponents of peritoneal lavage reason that contamination is both removed and diluted by lavage volumes greater than 10 L, additionally, by adding antibiotics bacterial pathogens can be specifically targeted. One group has suggested that lavage with volumes of approximately 20 L reduces infectious complications in blunt traumatic small bowel perforation[32]. However, its application with or without antibiotics in abdominal sepsis is largely unsubstantiated; at this time there is minimal evidence in the literature to support its use[33,34].

### Debridement

Debridement is essential for removal of foreign bodies, fecal matter, hematoma, and infected or necrotic tissue. The necessity to remove fibrin deposits is controversial. One early study showed improved postoperative courses with fewer continued infections; however, more recent studies have shown no benefit to this strategy[35,36].

### Definitive management

Definitive management involves restoration of anatomy and function. While staged procedures were once the standard, single stage procedures with primary anastomoses have become accepted as both safe and cost effective in the stable patient[37]. Still, establishing bowel continuity may need to be delayed in patients who are unable to tolerate a lengthy procedure or have inadequate capacity for tissue healing[38].

#### Specific Surgical Pathologies

### Appendicitis

Acute appendicitis is the most common intra-abdominal surgical emergency[19]. Lifetime risk is approximately 7-9%[39]. Currently, imaging is recommended for all patients suspected of having appendicitis except men under 40 years of age[40]. Generally, CT scan is the accepted imaging modality, however, ultrasound may have a role in women at risk for other pelvic pathologies, in pregnancy and in children[41]. The sensitivity and specificity of CT scan in the diagnosis of acute appendicitis are 87-100% and 91-98%, respectively[42,43]. Ultrasound is very user dependent, and results can be affected by patient body habitus, however overall sensitivity is 76-96% and specificity is 91-100%[44]. Ultrasound, with its decreased cost, lack of ionizing radiation and ability to assess ovarian pathology, has been the preferred initial imaging modality in children[45-47]. However, CT should be used in children when the initial ultrasound is negative or non-diagnostic and there is a high clinical suspicion for appendicitis[45,48]. Ultrasound is also the initial imaging procedure of choice in pregnant women, however, the appendix is visualized only 13-50% of the time. Magnetic resonance imaging (MRI) is an emerging imaging modality for cases of appendicitis in pregnancy with non-visualization of the appendix on ultrasound. Its sensitivity and specificity are 100% and 93.6%, respectively[49].

Though acute appendicitis is a very common entity, its management still contains areas of controversy including the role of laparoscopy, and the emerging role of medical management. These decisions can be complicated by the presence of an abscess or phlegmon.

Surgical management of acute appendicitis has been the gold standard of treatment for decades. However, many groups have proposed that in select patients, acute uncomplicated appendicitis can be treated with antibiotics alone. Initial success rates for conservative management of acute appendicitis range from 88-95%; however, recurrence is common, occurring in up to 35% of cases[50].

Both laparoscopic and open appendectomy are safe and effective. In large reviews, laparoscopic appendectomy has been associated with fewer surgical site infections, less pain, shorter hospital stays, and more rapid return to normal activity[51]. Common disadvantages found include increased cost and longer operative times[52,53]. Additionally, laparoscopy has been associated with increased risk of intra-abdominal abscess formation, especially in the presence of perforation or gangrene. In these cases, open surgery may be preferred[54]. Ultimately, the differences in outcomes between laparoscopic and open appendectomy are largely equivocal and the decision should be based on available technology and surgeon expertise, with increased consideration for laparoscopy in young female or obese patients[51,55,56].

Management of patients presenting with abscess or phlegmon is conservative, with antibiotics and drainage initially. Traditionally this has been followed by interval appendectomy. However, recently the need for interval appendectomy has been questioned. Controversy primarily surrounds the issues of recurrence and potential for malignancy. In a large review the recurrence rate was 7.4% and the risk of malignancy 1.2%[57]. This is in accord with similar studies that conclude that in asymptomatic patients, interval appendectomy has no advantages over a thorough work up for inflammatory appendiceal masses[58,59].

### Gastroduodenal perforation

After bleeding, perforation is the second most common complication requiring emergent operative intervention in peptic ulcer disease[60,61]. *Helicobacter pylori *infection is the most common cause of gastric and duodenal ulcers. Since the development of treatments for *H. pylori*, its prevalence in the United States has decreased. However, prevalence of gastric and duodenal ulcers has remained the same[62].

Previously, ulcer perforation was treated by excision and vagotomy. However, with antimicrobial eradication and anti-secretory pharmaceuticals, *H. pylori *positive ulcer recurrence has been significantly reduced[63]. As a result, the current standard of care is simple ulcer excision and primary repair of the bowel defect, or omental patch and subsequent *H. pylori *eradication, with little or no role for anti-secretory ulcer surgery[61,64].

Both open and laparoscopic approaches are reasonable options for treatment of perforated peptic ulcers. Laparoscopic surgery is associated with significantly less pain, but downfalls include longer operative times, and potentially inadequate repair of large perforations. Comparisons of sutured versus non-sutured repair with fibrin glue plug reveal that both are safe[65].

Conservative management has also been proposed as a safe option for management of contained or sealed gastroduodenal perforations. One randomized study showed similar morbidity and mortality for operative and conservative approaches; however, conservative treatment was associated with longer hospital stays and increased failure in patients over 70 years old[66]. Similarly, another author suggests that patients less than 40 years old and not on NSAIDS are the most likely to be infected with *H. pylori *and therefore, the most likely to benefit from non-operative therapy[67]. Alternatively, one group suggests that non-operative therapy can be guided by documented self-sealing on gastroduodenogram[68].

### Diverticulitis

Diverticular disease has increased since the turn of the 20th century[69]. The prevalence of diverticular disease among the general population is unknown, but at autopsy more than 50% of people over 80 years old are affected[70]. The lifetime prevalence of diverticulitis among patients with diverticulosis is 10-25%[69].

The standard treatment for uncomplicated diverticulitis is bowel rest and antibiotics. Most patients with uncomplicated diverticulitis respond to conservative management. Two studies found that patients who did not respond to antibiotics within 48 hours were more likely to require prolonged hospital stays for IV antibiotics and/or surgical intervention[71,72].

Diverticulitis can be complicated by phlegmon, abscess, or free perforation and is generally classified according to modified Hinchey criteria[73]. Approximately 15-20% of cases are associated with abscesses[74]. In cases of uniloculated abscess, the initial treatment is usually percutaneous drainage; although, in small abscesses (< 4 cm), antibiotics have been used as a primary treatment with success rates comparable to drainage[75,76]. When percutaneous drainage is performed it has success rates of up to 90%[77]. Of importance, the success of percutaneous drainage also seems to be dependent upon location. Ambrosetti and colleagues found that compared to mesocolic abscesses, pelvic abscesses were more aggressive, needed earlier drainage, and were more likely to require surgery[78].

Traditionally, patients who present with an abscess or phlegmon then undergo elective surgery to avoid the high risk of recurrence and further complications[71,73]. Recently though, some have begun to question the need for operative therapy when initial management with percutaneous drainage and antibiotics is successful[79]. Two authors have found that perforation, which is the most common cause of mortality in complicated diverticulitis, is more likely to be the initial presentation of disease, rather than a manifestation of recurrence[79,80]. They concluded that abscesses in complicated diverticulitis might then be adequately managed with antibiotics and drainage alone.

While conservative management may be appropriate in uniloculated abscesses, timely initial operative management is required for cases in which abscesses are large, multiloculated, or inaccessible, as well as in cases of free perforation, or diffuse peritonitis. Acute diverticulitis is complicated by free perforation in approximately 1.5% of episodes[81]. The standard procedure in cases of peritonitis is a Hartmann's procedure. However, the Hartmann's procedure is associated with significant morbidity and mortality, and while it can be reversed in 3-6 months, 30-70% of patients never undergo reversal[82-86]. Recently, it has been suggested that primary resection and anastomosis should be preferred[83,86,87]. Finally, laparoscopic resections for complicated diverticulitis have also been shown to be safe; and, in spite of longer operative times, they are associated with fewer major complications, less pain, and shorter hospital stays[88].

## Antibiotic Therapy

Surgery is the definitive treatment for complicated IAI, but systemic antibiotic therapy is a necessary adjunct. The role of antibiotics in this setting is prevention and treatment of hematogenous spread of infection and reduction of late complications[89]. Treatment should be initiated as soon as a diagnosis is suspected, and within an hour in the case of severe sepsis[22]. Antibiotic choice should depend on the most likely source of infection, immune status of the patient, and the likelihood of opportunistic or resistant organisms.

In general, the gastrointestinal tract is sterile in the stomach and duodenum, with enteric gram negatives in the proximal small bowel, and anaerobes populating the distal ileum and colon[7]. Table 1 lists the expected organisms according to source of contamination.

In cases where the source is known, antimicrobial selection can target site-specific organisms. When the source is not known, choice of antimicrobial regimen and duration of treatment should be guided by patient risk. Risk, in this context, is intended to describe risk for failure of treatment, and risk assessment allows for proper selection of narrow versus broad-spectrum antibiotics. High versus low risk is determined primarily by patient physiology and underlying medical conditions (Table 2). Health care-associated infections, APACHE II score > 15, advanced age, organ dysfunction, poor nutritional status, immunosuppression and presence of malignancy are all associated with a high risk of treatment failure[5,12].

**Table 2:** Risk factors for poor outcomes

Factors associated with high risk for poor outcomes	
Pre-existing factors	Disease specific
Poor nutritional status	APACHE II score ≥ 15
Presence of malignancy	Delay in initial intervention > 24 hours
Organ dysfunction	Inadequate source control
Immunosuppression	Prolonged pre-operative hospital stay
	Prolonged pre-operative antibiotics

Adapted from Weigelt JA, Solomkin, Wacha [4,12,40,109].

Without identifiable risk factors, an IAI is considered low risk and can be treated with narrow-spectrum antibiotics directed toward anaerobic and gram-negative organisms[7]. In low risk infections, cultures are generally considered unnecessary. Even if cultures are obtained and show resistant organisms, there is no need to alter antimicrobial therapy according to culture results if there is an adequate clinical response[5]. Table 3 lists antibiotic regimens deemed appropriate for low risk patients by the Surgical Infection Society (SIS).

**Table 3:** Risk stratified antibiotic recommendations

	Low Risk	High Risk
**Single Agent**	Cefoxitin	Imipenem-cilastatin
	Ertapenem	Meropenem
	Moxifloxacin	Doripenem
	Ticarcillin	Pipercillin-tazobactam
	Tigecycline	
**Combination**	Cefazolin	Cefepime
	Cefuroxime	Ceftazidime
	Ceftriaxone	Ciprofloxacin
	Cefotaxime	Levofloxacin
	Ciprofloxacin	+Metronidazole
	Levofloxacin	
	+Metronidazole	

Adapted from Solomkin[4,5] (Infectious Diseases Society of America Guidelines).

High-risk patients require the use of broad-spectrum antibiotics with anticipation of resistant organisms (see Table 3). Additionally, in high-risk patients attention should be given to the antibiograms of the particular institution, with initial antibiotic choice tailored to the risk of methicillin or vancomycin resistant organisms, and extended spectrum beta lactamase producers. Compared to patients initially treated with broad-spectrum antibiotics, patients who receive inadequate empiric treatment have longer hospital stays, higher rates of postoperative abscesses and re-operation, and increased mortality[90,91]. Furthermore, changing regimens in response to cultures that display resistance does not improve outcomes[90]. Therefore, the use of broader-spectrum agents from the outset appears crucial to optimizing outcomes in high-risk patients. While cultures do not alter outcomes in high risk patients, it is recommended that cultures be obtained in this group in order to de-escalate antibiotic therapy to avoid increasing resistance[40].

### Infections that Require Special Consideration

#### MRSA

Though an uncommon cause of IAI, *MRSA *deserves special consideration. Treatment often includes vancomycin, which has a low bactericidal activity and achievable tissue concentrations of the drug may not meet the minimum inhibitory concentration (MIC)[92]. As a result, these infections may require longer courses of antimicrobial therapy[89]. Continuous infusion of vancomycin may be a solution to this problem. In addition, newer antibacterials such as linezolid, tigecycline, ertapenem, and moxifloxacin are also promising, and have demonstrated non-inferiority in several studies of IAI[40,92-95].

#### Enterococcus

The use of antibiotic therapy for *Enterococcus *in IAI is controversial. *Enterococcus *can often be isolated from IAI, and is associated with increased risk of treatment failure and higher mortality[96,97]. However, outcomes in these patients have shown to be independent of antibiotic coverage for enterococcus[97,98]. Currently, the general consensus regarding enterococcal coverage is that community-acquired infections require no coverage, however ampicillin, or vancomycin should be added to cover the following high risk patient groups: 1) patients in septic shock who have received prolonged treatment with cephalosporins or other antibiotics that select for Enterococcus, 2) immunocompromised patients, 3) patients with prosthetic heart valves, or other intravascular prosthetic devices, or 4) patients with health care associated/recurrent intra-abdominal infection[40,99]. Finally, vancomycin resistant enterococcal (VRE) infections occur in patients who are immunocompromised, previously colonized with VRE or treated with vancomycin[100]. In these circumstances VRE should be suspected and treated with alternatives such as linezolid, tigecycline, or daptomycin. In the absence of these risk factors, specific coverage for VRE is not recommended[40].

#### Candida

Candida is similar to *Enterococcus*, in that isolation of *Candida *from intra-abdominal cultures is associated with increased mortality, but anti-fungal treatment has not been shown to alter this risk[101]. Therefore, fungal coverage is unnecessary unless the patient is immunocompromised, has a severe IAI with *Candida *grown from intra-abdominal cultures, or has perforation of a gastric ulcer while on acid suppressive medications[102]. Fluconazole is an appropriate initial choice for *Candida albicans *peritonitis. However, increasingly, non-albicans *Candida spp*., with resistance to commonly used anti-fungals are responsible for candidemia[103,104]. Studies have shown that echinocandins are both safe and effective in the treatment of invasive candidiasis. Therefore, in critically ill patients echinocandins, such as caspofungin or echinofungin, should be considered for primary treatment[102,104]. Required treatment duration for *Candida *peritonitis is 2-3 weeks[102].

### Duration of Treatment

Because resistant organisms have been linked to imprudent use of antibiotics, it is important to limit the duration of antimicrobial treatment[105]. Previously, studies have suggested limiting treatment duration for IAI by discontinuing antibiotics when fever and leukocytosis have resolved, and the patient is tolerating an oral diet[106]. More recently, it has been suggested that fixed duration treatment has similar efficacy[107]. The Surgical Infection Society (SIS) recommends that duration for complicated abdominal infections should be limited to 4-7 days, and may be discontinued sooner in the absence of clinical signs of infection[40]. In addition, once patients are able to tolerate oral intake, antibiotic therapy can be transitioned to oral dosing for the remainder of their treatment without increased risk of failure[108]. Suggested oral regimens for patients in whom resistance is not a concern are listed in Table 4. Of note, lack of resolution of clinical signs of infection after 7 days of antibiotics implies failed source control, tertiary peritonitis, or new infection. Further diagnostic work up including labs, cultures and imaging to look for new or continued sources of infection is essential, and should be accompanied by further surgical intervention if warranted[2].

**Table 4:** Recommended oral regimens

Oral regimens	
Single agent	Double agent
Amoxicillin-clavulinic acid	Moxifloxacin/Ciprofloxacin/Levofloxacin +Metronidazole
	Oral cephalosporin +Metronidazole

Adapted from Solomkin[4] (Guidelines by the Surgical Infection Society and the Infectious Diseases Society of America).

Finally, we must consider patients with acute IAI, for which prompt source control is achieved. In cases where adequate source control is accomplished within 12-24 hours, less than 24 hours of antibiotic treatment is necessary (Table 5). Antibiotic choice in these instances should generally be guided by the aforementioned recommendations for low risk infections.

**Table 5:** Conditions requiring less than 24 hours of antibiotic therapy

Conditions requiring < 24 hours of antibiotic therapy	
Trauma	Non-trauma
Bowel injuries due to blunt trauma repaired in < 12 hours	Acute stomach and proximal jejunal perforation in the absence of malignancy or acid reducing therapy repaired in < 24 hours
Bowel injuries due to penetrating trauma repaired in < 12 hours	Intraoperative contamination repaired in < 12 hours
	Acute appendicitis/cholecystitis without perforation, abscess or local peritonitis

Adapted from Solomkin[4](Guidelines by the Surgical Infection Society and the Infectious Diseases Society of America).

## Conclusion

Successful management of IAI is multi-factorial. Source control is of primary importance. Prompt and judicious antibiotic therapy is also necessary. Appropriate antibiotic therapy requires patient risk stratification. Duration of antibiotic treatment should be limited to one week, followed by re-evaluation and intervention as needed.

## Competing interests

The authors declare that they have no competing interests.

## Authors' contributions

NL participated in literature review and preparation of the manuscript. LK participated in literature review and preparation of the manuscript. RC conceived and designed this review and participated in creation of the outline, and preparation of the manuscript. All authors read and approved the final manuscript.
